# Epidemiology of Monoclonal Gammopathies in Sub‐Saharan Africa: A Systematic Review and Meta‐Analysis of MGUS and Multiple Myeloma

**DOI:** 10.1111/tmi.70066

**Published:** 2026-01-28

**Authors:** Iyanuloluwa S. Ojo, Olurotimi John Badero, Toluwalope Olawale Oluwalana, Nicholas Aderinto, Faridat Ibidun, Onur Oral, Emmanuel Oyesiji, Evelyn Faith Ogungbemi

**Affiliations:** ^1^ Nuffield Centre for International Health and Development, Leeds Institute of Health Sciences University of Leeds Leeds UK; ^2^ Department of Cardionephrology Cardiac Renal & Vascular Associates Jackson Mississippi USA; ^3^ Mersey and West Lancashire NHS Trust Blackpool UK; ^4^ Ladoke Akintola University of Technology Ogbomoso Nigeria; ^5^ Noble's Hospital Douglas Isle of Man; ^6^ Faculty of Health and Sports Sciences Ege University Izmir Turkey; ^7^ College of Medicine, University of Ibadan Ibadan Nigeria; ^8^ Rostov State Medical University Rostov‐on‐Don Russia

**Keywords:** epidemiology, HIV, meta‐analysis, monoclonal gammopathy of undetermined significance, multiple myeloma, prevalence, renal failure

## Abstract

**Background:**

Monoclonal gammopathy of undetermined significance (MGUS) and multiple myeloma (MM) are reported to occur more frequently in Black populations, particularly African Americans. However, despite Sub‐Saharan Africa (SSA) being home to the largest Black population globally, epidemiological data on MGUS and MM in the region remain scarce.

**Methods:**

We conducted a systematic review and meta‐analysis of published studies from SSA to estimate the pooled prevalence of MGUS and MM, regional variations, survival outcomes and key clinical complications, including HIV‐associated MM, renal failure, anaemia and hypercalcaemia.

**Results:**

Forty‐five studies were included in this systematic review and meta‐analysis, with eight on MGUS and 37 on MM. The pooled prevalence of MGUS in SSA was 3.1% (95% CI: 0.8%–12.0%), while MM prevalence was 7.8% (95% CI: 5.6%–10.4%). MM burden was highest in Central Africa and lowest in West Africa, whereas MGUS prevalence peaked in Southern Africa. The pooled mean survival for MM was 34.7 months; 1‐year and 5‐year overall survival rates were 46.0% and 20.7%, respectively. HIV significantly modified MM outcomes: the pooled prevalence of MM among people living with HIV was 7.2% (95% CI: 1.9%–24.2%), and mean survival was markedly shorter in HIV‐positive patients (9.9 months) compared with HIV‐negative patients (36.7 months). Renal failure was highly prevalent, affecting 30.1% (95% CI: 23.5%–37.6%) of MM patients at diagnosis.

**Conclusion:**

MM and MGUS impose a substantial and under‐recognised burden in SSA, with poorer survival outcomes compared with global averages. The high rates of delayed presentation, limited diagnostic capacity, absence of routine plasma cell disorder screening and restricted access to modern therapies likely contribute to the disparities observed. Strengthening diagnostic infrastructure and improving early detection and treatment pathways are urgently needed to advance equity in myeloma care across SSA.

## 1 Introduction

Multiple myeloma (MM) accounts for the third highest incidence rate among all haematological malignancies in the world, and it is currently incurable, with a median overall survival rate of 38.8 months (3.2 years) in patients who could not afford the autologous stem cell transplantation [1, 2].

MM is a plasma cell neoplasm characterised by proliferation of plasma cells within the bone marrow, associated with characteristic calcium elevation, renal failure, anaemia and bone lesion (CRAB) features, ultimately leading to progressive end organ damage [3, 4]. It is a spectrum from the monoclonal gammopathy of undetermined significance with no CRAB features to smouldering MM to finally symptomatic MM [4, 5].

In Africa, MM is relatively underdiagnosed when compared to other malignancies [6]. The limitation of diagnostic hemopathology such as the unavailability of cytogenetic and molecular techniques, especially the fluorescence In Situ Hybridization technique, also hinders the diagnosis of MM in Africa [7].

This needs to be addressed given the higher incidence of MM among Americans of African descent, with a prevalence of 8% compared to 3.8% among White Americans. The same pattern is observed in the MM precursor, MGUS, where a 16‐year study showed the age adjusted prevalence in Black Americans being three times higher than Whites. Thokerunga et al., however, attributed this disparity to the lack of routine screening for individuals over 40 years of age in Africa, as well as low awareness among primary care clinicians regarding the signs and symptoms of MM [6, 8, 9].

The disparity in the prevalence of MM and MGUS between Black and White populations has been the subject of numerous investigations [10]. Genetic susceptibility has been considered a key factor, with the Molecular and Genetic Epidemiology (iMAGE) study of MM having an overwhelming higher odds ratio for family history associated MM in Blacks 20.9 (95% CI: 2.6 to 168) than in Whites 2.0 (95% CI: 0.8 to 5.0) [10, 11]. Socioeconomic status was also surprisingly considered to be responsible for the disparity of MM [10, 12]. Baris et al. in 2000 found that a low occupation‐based socioeconomic status score was significantly associated with MM prevalence [12]. Obesity was also found to be associated with the high prevalence of MM in Blacks [10, 13, 14].

Despite this overwhelming risk in Blacks, the largest increase in incidence of MM from 1990 to 2016 in a global epidemiologic study by Cowan AJ et al. was recorded in East Asia [15]. Africa was not in the top three regions with the highest age‐standardised incidence and death rates for MM. Even in absolute numbers, Western Europe peaked the chart [15]. Sub Saharan Africa was recorded as one of the regions with the lowest age‐standardised incidence rate despite the region greatly affected by proven MM risk factors such as Low socioeconomic status, genetic susceptibility in Blacks and even recently, obesity [15].

It is therefore pertinent to understand the current epidemiologic trends of MM and its precursor, MGUS in Africa which will serve as a bedrock to understanding how to approach MM screening and management in Sub‐Saharan Africa.

### 1.1 Aim

To comprehensively characterise the epidemiology, disease presentation and survival outcomes of monoclonal gammopathy of undetermined significance (MGUS) and MM in Sub‐Saharan Africa through a systematic review and meta‐analysis.

### 1.2 Objectives

- To estimate the pooled prevalence of MGUS and MM and assess regional and country‐level variations across Sub‐Saharan Africa.
- To quantify the burden of advanced disease and key clinical complications at diagnosis, including Stage III MM, renal failure, anaemia and hypercalcaemia.
- To synthesise survival outcomes such as mean survival duration, and 1‐year and 5‐year overall survival among MM patients in the region.
- To evaluate the impact of HIV infection on MGUS and MM epidemiology, including pooled HIV prevalence among MM patients, MGUS–HIV associations and survival differences.

## 2 Materials and Methods

### 2.1 Literature Search and Data Extraction

This systematic review and meta‐analysis adhered to the PRISMA reporting guidelines [16]. A comprehensive search was conducted in PubMed, Cochrane Library and Google Scholar for studies published between 1 January 2002 and 1 July 2025. An additional search was performed in African Journals Online (AJOL) between 25 October 2025 and 11 November 2025. The search strategy combined Medical Subject Headings (MeSH), including exploded terms, with free‐text keywords. Search approaches were customised for each database to ensure maximal coverage. Core concepts included “multiple myeloma in Sub‐Saharan Africa,” “monoclonal gammopathy of undetermined significance (MGUS) in Sub‐Saharan Africa,” “prevalence,” “mortality” and “HIV.” Reference lists of included studies and citation tracking through Google Scholar were also used to identify additional eligible records.

Only studies published in English or French were included, as these are the most widely used languages in Sub‐Saharan Africa. Inclusion criteria were:

- studies reporting prevalence of MM or MGUS in Sub‐Saharan Africa, either alone or in combination with other hematologic malignancies, provided that prevalence and/or mortality were assessed using validated or piloted tools; and
- studies diagnosing monoclonal gammopathies using standard laboratory techniques, such as serum protein electrophoresis (SPEP), urine protein electrophoresis and immunofixation, alongside clinical and biochemical criteria consistent with International Myeloma Working Group (IMWG) guidelines.

For multiple myeloma, eligible studies required evidence of clonal plasma cell proliferation supported by CRAB features or myeloma‐defining events.

For MGUS, included studies were required to define the condition as serum M‐protein < 30 g/L, < 10% clonal plasma cells in bone marrow and absence of end‐organ damage. Cytogenetics and FISH testing were not required for inclusion, as these are primarily used for risk stratification, not initial diagnosis.

Exclusion criteria were non‐English/French studies, case reports, narrative reviews, systematic reviews, qualitative studies and those not meeting diagnostic criteria.

Two reviewers independently screened titles, abstracts and full texts for eligibility. A third reviewer evaluated all full‐text decisions to ensure consistency. Data extraction was performed using a standardised WPS spreadsheet by one reviewer (ISO), capturing study design, sample size, prevalence estimates, demographic characteristics, HIV prevalence and clinical/biochemical parameters (e.g., presenting symptoms, renal involvement, haemoglobin levels, Durie–Salmon and ISS stage, serum calcium and ESR levels).

Methodological quality was assessed using the Joanna Briggs Institute (JBI) Critical Appraisal Checklist for Studies Reporting Prevalence Data [17, 18]. This tool evaluates sample frame adequacy, sampling methods, sample size sufficiency, clarity of study subjects and setting, analytical coverage, diagnostic methods, measurement reliability, appropriateness of statistical analysis and response rates. Studies were categorised as low (0–3), moderate (4–6), or high (7–9) quality out of a maximum possible score of nine. Discrepancies between reviewers were resolved through consensus.

### 2.2 Analytical Methods and Meta‐Analysis

All analyses were performed using R version 4.4.3, primarily with the metafor and meta packages. Prevalence estimates for MM and MGUS were pooled using a random‐effects model because of substantial clinical and methodological heterogeneity across studies. Study‐level proportions were logit‐transformed (PLOGIT) and aggregated using inverse‐variance weighting, with final estimates back‐transformed to percentages. Heterogeneity was assessed using Cochran's *Q*, *I* ^**2**^ and *H* statistics, each with 95% confidence intervals.

To explore potential modifiers of prevalence, predefined subgroup analyses were conducted by publication year, mean age at diagnosis, geographic region, country, study duration and methodological quality. The extremely large Madagascar dataset (*n* = 11,374) was excluded from subgroup models to avoid distortion of pooled estimates.

Publication bias was evaluated using funnel plot asymmetry and Egger's regression test. When significant bias was identified, the non‐parametric trim‐and‐fill method was applied to generate adjusted pooled estimates and identify potentially missing studies.

For survival outcomes, a sample‐size–weighted pooled mean was calculated because most studies did not report standard deviations. Additional pooled analyses examined 1‐year and 5‐year overall survival, and subgroup comparisons were performed for studies reporting HIV prevalence, renal failure prevalence, anaemia burden and hypercalcaemia. Where relevant, continuous study‐level covariates (e.g., renal‐failure prevalence, haemoglobin levels) were converted into high versus low categories using median or clinically meaningful thresholds to allow comparable subgroup pooling.

Associations between MM and HIV were examined through pooled prevalence, while the association between MGUS and HIV was estimated using pooled odds ratios from the two available studies.

An exploratory linear regression and Spearman correlation were conducted to assess the relationship between MGUS and MM prevalence. Owing to substantial heterogeneity in study designs, populations and diagnostic approaches, the resulting estimates lacked interpretability and were excluded from the final analysis.

## 3 Results

**FIGURE 1:**
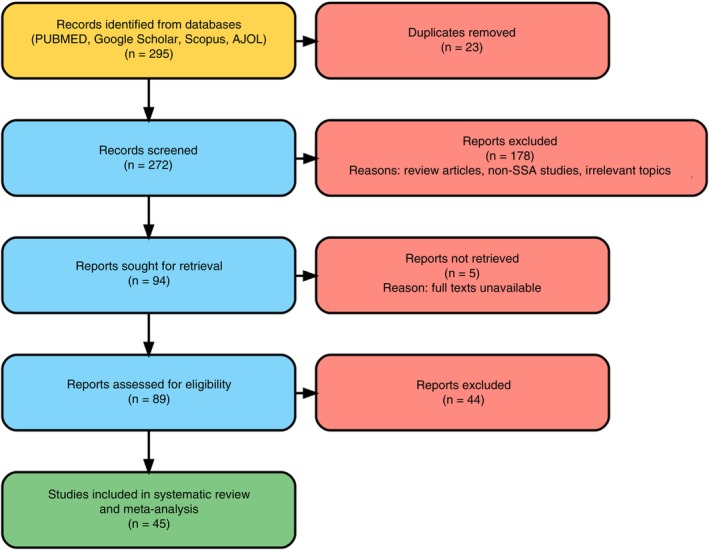
PRISMA flow chart of literature search results and study selection.

Databases search yielded 295 records. After inclusion criteria were employed, 45 studies, 37 on MM and 8 on monoclonal gammopathy of undetermined significance (MGUS) were extracted and included in the systematic review and meta‐analysis as shown in Figure 1.

The characteristics of the study can be found in Table 1. Thirty‐seven studies were on MM while the other eight studied MGUS. Most of the studies are retrospective cohort studies (*n* = 30); there are 3 prospective cohort studies, 11 cross‐sectional studies and 1 longitudinal study. Nineteen of the studies were carried out in Nigeria, eight from South Africa, three from Ghana, three from Cameroon, two from Kenya, two from Tanzania and one each from DR Congo, Mali, Madagascar, Ethiopia, Eritrea, Togo, Uganda and Eswatini, making it a total of 23 studies from West Africa, five from Central Africa, seven from East Africa and nine from South Africa. The study with the highest sample size was carried out in Madagascar (Randrianarisoa et al. 2023) with a sample size of 11,374 while the lowest was in Nigeria (Salawu et Durosinmi 2005) with a sample size of 27. Based on Joanna Briggs Institute (JBI) Critical Appraisal Checklist for Studies Reporting Prevalence, there are 26 studies of high methodological quality and 19 studies of moderate methodological quality.

**TABLE 1:** Characteristics of studies included in the systematic review and meta‐analysis.

Author, year	MM/MGUS	Country	Study design	Sample size	Methodological quality
Nnonyelum [19]	Multiple myeloma	Nigeria	Retrospective cohort	135	Moderate (4/9)
Omoti et Omoemu [20]	Multiple myeloma	Nigeria	Prospective cohort	30	High (7/9)
Salawu et Durosinmi [21]	Multiple myeloma	Nigeria	Retrospective cohort	27	Moderate (6/9)
Acquah [22]	Multiple myeloma	Ghana	Retrospective cohort	169	High (8/9)
Okello [23]	Multiple myeloma	Uganda	Retrospective cohort	217	High (7/9)
Chili [24]	Multiple myeloma	South Africa	Retrospective cohort	135	High (8/9)
Baxter [25]	Multiple myeloma	South Africa	Retrospective cohort	601	Moderate (6/9)
Manyega [26]	Multiple myeloma	Kenya	Retrospective cohort	221	High (8/9)
Nkanga [27]	Multiple myeloma	DR Congo	Prospective cohort	105	Moderate (5/9)
Mbanya [28]	Multiple myeloma	Cameroon	Retrospective, cross sectional	172	Moderate (6/9)
Randrianarisoa RMF [29]	Multiple myeloma	Madagascar	Retrospective cohort	11,374	High (8/9)
Babatunde [30]	Multiple myeloma	Nigeria	Retrospective cohort	370	Moderate (6/9)
Dirisu [31]	Multiple myeloma	Nigeria	Longitudinal	73	Moderate (6/9)
Raza [32]	Multiple myeloma	Tanzania	Cross sectional	76	High (8/9)
Abdullah [33]	Multiple myeloma	South Africa	Retrospective, cross sectional	374	High (8/9)
Dachi [34]	Multiple myeloma	Nigeria	Retrospective cohort	493	Moderate (6/9)
Egesie [35]	Multiple myeloma	Nigeria	Retrospective cohort	60	Moderate (6/9)
Onoja [36]	Multiple myeloma	Nigeria	Retrospective cohort	78	Moderate (6/9)
Out [37]	Multiple myeloma	Nigeria	retrospective cohort	328	High (9/9)
Dachi [38]	Multiple myeloma	Nigeria	Retrospective cohort	71	Moderate (6/9)
James [39]	Multiple myeloma	Nigeria	Retrospective cohort	90	Moderate (6/9)
Egesie [40]	Multiple myeloma	Nigeria	Retrospective cohort	330	High (8/9)
Omoti [41]	Multiple myeloma	Nigeria	Retrospective cohort	391	High (8/9)
Ugwu [42]	Multiple myeloma	Nigeria	Retrospective cohort	135	Moderate (6/9)
Kagu [43]	Multiple myeloma	Nigeria	Retrospective cohort	236	Moderate (6/9)
Diallo [44]	Multiple myeloma	Mali	Retrospective cohort	264	Moderate (6/9)
Ngalagou [45]	Multiple myeloma	Cameroon	Retrospective cohort	454	High (8/9)
Mbanya [28]	Multiple myeloma	Cameroon	Retrospective, cross sectional	172	High (7/9)
Ebrahim [46]	Multiple myeloma	Ethiopia	Cross sectional	228	High (8/9)
Wilson [47]	Multiple myeloma	South Africa	Retrospective cohort	120	High (8/9)
Leak [48]	Multiple myeloma	Tanzania	Cross sectional	209	Moderate (6/9)
Belai [49]	Multiple myeloma	Eritrea	Retrospective cohort	207	High (8/9)
Inamasu [50]	Multiple myeloma	South Africa	Retrospective cohort	1880	Moderate (6/9)
Kueviakoe [51]	Multiple myeloma	Togo	Retrospective cohort	469	High (7/9)
Okinda et Riyat [52]	Multiple myeloma	Kenya	Retrospective cohort	356	High (7/9)
Oelofse et Truter [53]	Multiple myeloma	South Africa	Retrospective cohort	3603	High (9/9)
Vachon [54]	MGUS	Nigeria	Cross‐sectional study	343	High (7/9)
Oyeyinka [55]	MGUS	Ghana	Retrospective cohort	222	Moderate (4/9)
Onwah [56]	MGUS	Nigeria	Cross‐sectional study	410	High (8/9)
Lee [57]	MGUS	South Africa	Cross‐sectional study	738	High (9/9)
Landgren [58]	MGUS	Ghana	Prospective cohort, nested case series	917	High (9/9)
Cicero [59]	MGUS	South Africa	Cross‐sectional study	386	High (9/9)
Cicero [60]	MGUS	Eswatini	Cross‐sectional study	515	High (9/9)
Otu et Ejikeme [61]	MGUS	Nigeria	Retrospective cohort	328	High (9/9)

### 3.1 Pooled Prevalence of Multiple Myeloma

**FIGURE 2:**
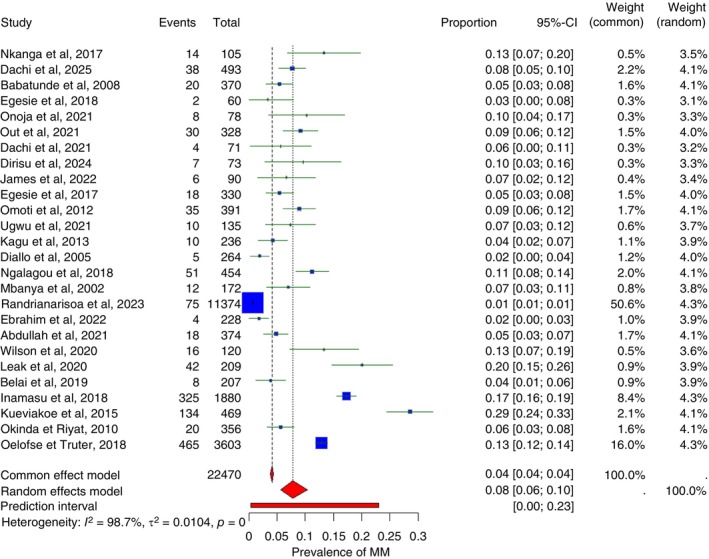
Forest plot of multiple myeloma in Sub‐Saharan Africa.

In this systematic review and meta‐analysis, the pooled prevalence of MM in Sub‐Saharan Africa was estimated at 7.8% (95% CI: 5.6%–10.4%), with wide variability across studies (Figure 2). Reported prevalence ranged from 0.7% in Madagascar (2023) to 28.6% in Togo (2015). There was substantial heterogeneity among studies, with an *I* ^**2**^ of 98.7%, indicating considerable variation not attributable to chance alone. Regionally, the pooled prevalence of MM was 7.6% (95% CI: 4.8%–11.0%) in West Africa, 10.4% (95% CI: 4.7%–17.9%) in Central Africa, 4.8% (95% CI: 0.5%–15.6%) in East Africa and 11.7% (95% CI: 4.2%–22.2%) in Southern Africa.

#### 3.1.1 Subgroup Analysis

As outlined in Table 2, subgroup analysis demonstrated that the prevalence of MM varied across different publication periods, age groups, regions, countries and study durations. By publication year, studies published between 2011 and 2019 had the highest pooled prevalence of 10.2% (95% CI: 5.7%–15.9%), followed by studies published during the COVID era (2020–2022) with a prevalence of 8.1% (4.5%–12.7%). Studies published before 2011 had a lower prevalence of 4.7% (1.7%–9.0%), while post‐COVID studies (2024–2025) showed a prevalence of 7.8% (0.9%–19.7%).

**TABLE 2:** Subgroup analysis result of the prevalence of multiple myeloma.

Subgroup	No. of studies	Pooled prevalence (95% CI)	Heterogeneity (*I* ^2^)
Publication year			
< 2011	4	4.7 (1.7–9.0)	66.7%
2011–2019	11	10.2 (5.7–15.9)	95.0%
2020–2022 (Covid Era)	8	8.1 (4.5–12.7)	86.3%
2023–2025 (Post Covid)	2	7.8 (0.9–19.7)	0.0
Mean age at diagnosis			
< 45	5	6.9 (5.9–8.9)	70.3%
45–55	5	7.2 (5.2–9.3)	0.0
> 55	4	10.2 (2.9–21.9)	89.4%
Region			
Western Africa	14	7.6 (4.8–9.5)	92.8%
Central Africa	3	10.4 (4.7–17.9)	44.8%
Eastern Africa	5	4.8 (0.1–15.6)	97.6%
Southern Africa	4	11.7 (4.2–22.2)	94.7%
Country			
Nigeria	12	6.8 (5.6–8.1)	20.4%
South Africa	4	11.7 (4.2–22.2)	94.7%
Duration of the study			
< 5 years	8	6.9 (3.0–12.1)	88.5%
5–10 years	10	9.0 (5.9–12.7)	93.0%
> 10years	7	7.2 (1.6–16.1)	98.9%
Methodological quality			
High	12	8.6 (5.1–12.9)	94.8%
Moderate	13	8.1 (5.2–11.6)	92.6%

Regarding age at diagnosis, MM was more common among individuals aged over 55 years, with a pooled prevalence of 10.2% (2.9%–21.9%), compared to 7.2% (5.2%–9.3%) in those aged 45–55 years and 6.9% (5.9%–8.9%) in those under 45 years.

Regional differences were also observed, with Southern Africa reporting the highest prevalence at 11.7% (4.2%–22.2%), followed by Central Africa at 10.4% (4.7%–17.9%), Western Africa at 7.6% (4.8%–9.5%) and Eastern Africa at 4.8% (0.1%–15.6%). Country‐level analysis revealed a higher prevalence in South Africa at 11.7% (4.2%–22.2%) compared to Nigeria at 6.8% (5.6%–8.1%).

Finally, studies with durations of 5–10 years showed the highest pooled prevalence of 9.0% (5.9%–12.7%), followed by studies lasting less than 5 years at 6.9% (3.0%–12.1%), while studies longer than 10 years had a prevalence of 7.2% (1.6%–16.1%). Overall, the analysis indicates variation in MM prevalence by time period, age, geography and study duration, with some evidence of higher prevalence in older populations and certain regions of Africa. Randrianarisoa et al. (2023) was excluded in subgroup analysis except for the regional analysis due to its extremely large sample size relative to other studies.

#### 3.1.2 Publication Bias

**FIGURE 3:**
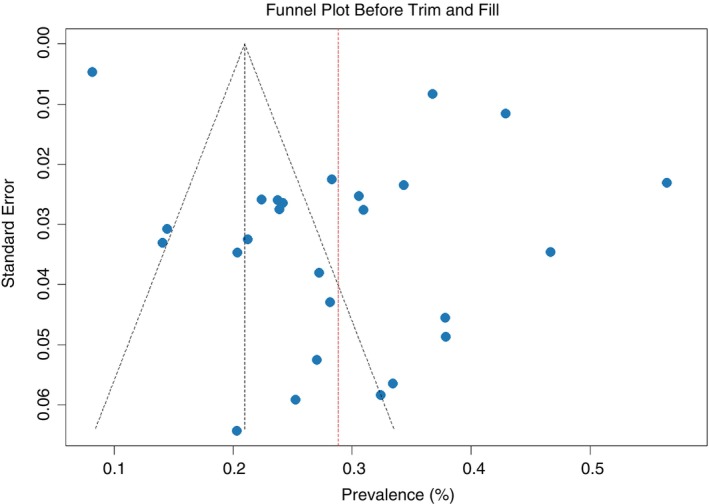
Funnel plot showing publication bias in this study (MM).

**FIGURE 4:**
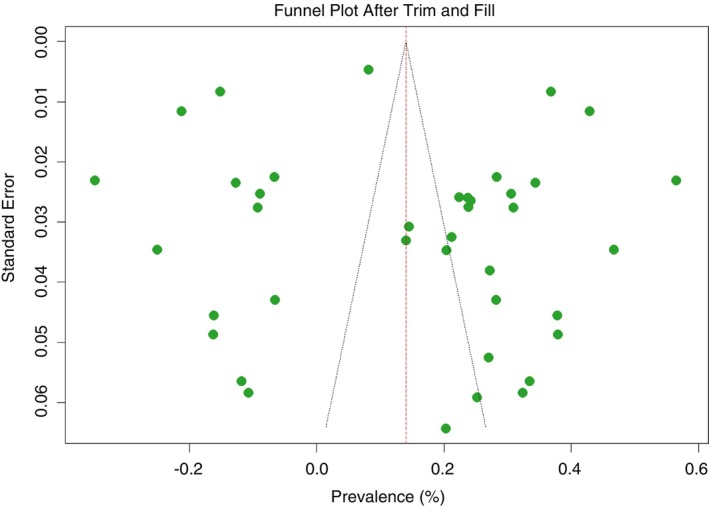
Funnel plot of this study after trim and fill analysis (MM).

Both the asymmetry of the funnel plot and the Egger's test signifies the existence of publication bias (*p* = 0.03). Therefore a non‐parametric trim and fill analysis was performed to reduce the publication bias. The Funnel plot before and after the trim and fill analysis are depicted in Figures 3 and 4, respectively.

#### 3.1.3 Non‐Parametric Trim and Fill Analysis

**FIGURE 5:**
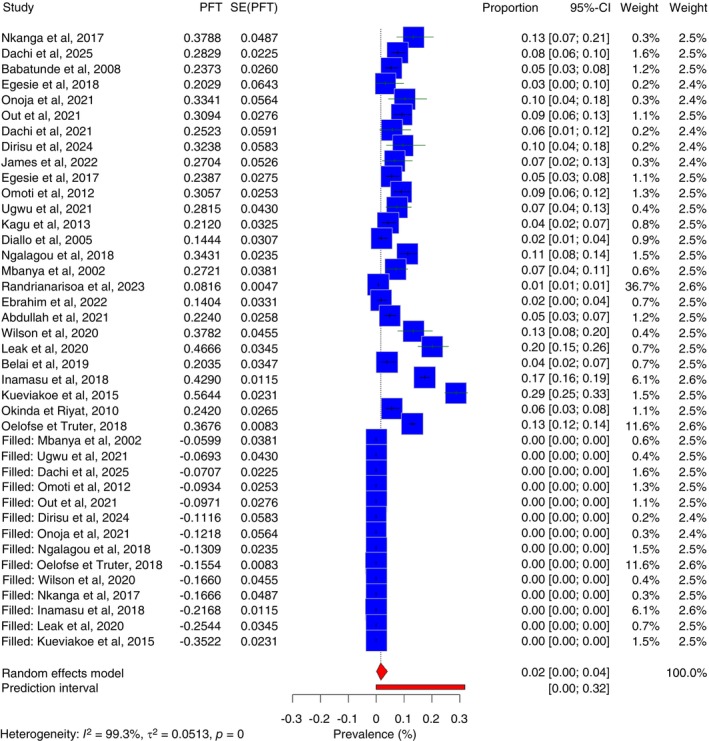
Forest plot of multiple myeloma in Sub‐Saharan Africa following a non‐parametric trim and fill analysis.

A non‐parametric trim and fill analysis was performed due to the detection of publication bias. Fourteen studies were added following trim and fill analysis using the random effects model *p*‐value, bringing it to a total of 40 studies. Following the addition of the 14 studies, the pooled prevalence of MM was revised to 15.6% (95% CI [0.2%–40.8%]) as shown in Table 3. A forest plot of MM in Sub‐Saharan Africa following trim and fill analysis is also shown in Figure 5.

**TABLE 3:** Pooled estimate of multiple myeloma prevalence in sub Saharan Africa before and after trim and fill analysis.

Studies	Pooled estimate	95% CI	Number of studies
Before trim and fill analysis	7.8%	5.6%–10.4%	27
After trim and fill analysis	15.6%	0.2%–40.8%	40

### 3.2 Pooled Prevalence of MGUS in Sub‐Saharan Africa

**FIGURE 6:**
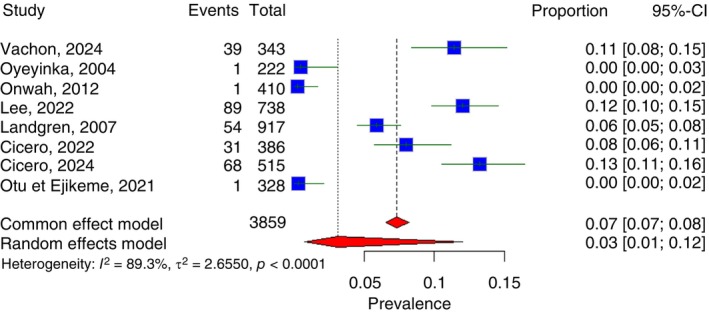
Forest plot of MGUS in Sub‐Saharan Africa.

In this systematic review and meta‐analysis, the pooled prevalence of monoclonal gammopathy of undetermined significance (MGUS) in Sub‐Saharan Africa was estimated at 3.1% (95% CI: 0.8%–12.0%) (Figure 6). The prevalence varied widely across studies, ranging from 0.2% in Lagos, Nigeria (2012) to 13.2% in Mbabane, Eswatini (2024). There was substantial heterogeneity among studies, with an *I* ^2^ of 89.3%, indicating considerable variation not attributable to chance alone. Regionally, the pooled prevalence of MGUS was 1.3% (95% CI: 0.1%–11.7%) in West Africa and 11.2% (95% CI: 0.7%–17.5%) in Southern Africa.

#### 3.2.1 Publication Bias

**FIGURE 7:**
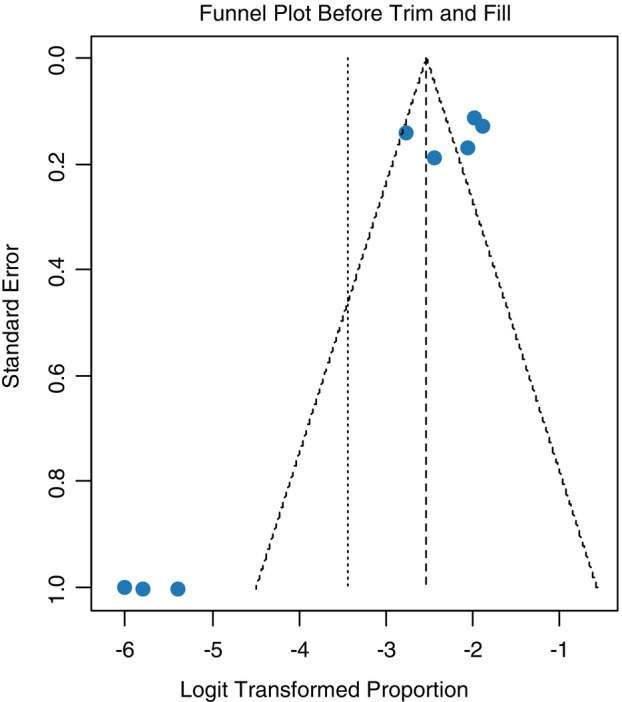
Funnel plot showing publication bias in this study (MGUS).

Both the asymmetry of the funnel plot and the Egger's test signify the existence of publication bias. Therefore, a non‐parametric trim and fill analysis was performed to reduce the publication bias (Figure 7).

#### 3.2.2 Non‐Parametric Trim and Fill Analysis

**FIGURE 8:**
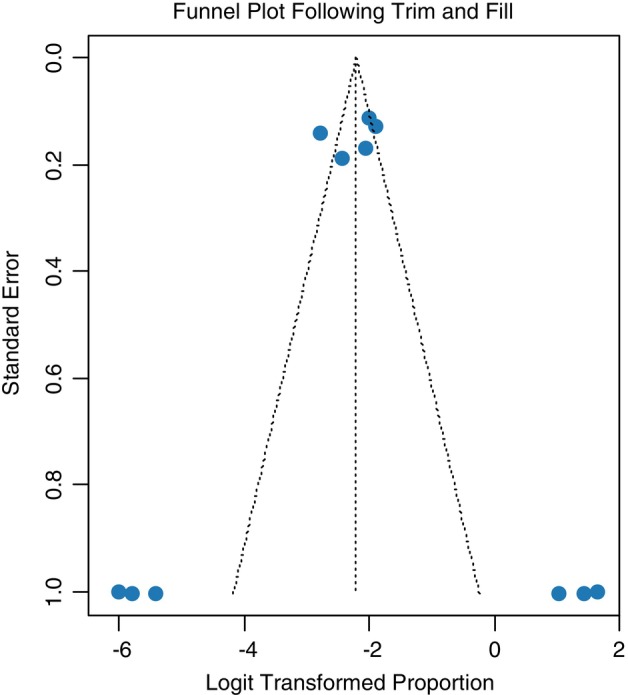
Funnel plot of this study after trim and fill analysis (MGUS).

A non‐parametric trim and fill analysis was performed due to the detection of publication bias (Figure 8). Three studies were added following trim and fill analysis using the random effects model *p* value, bringing it to a total of 11 studies. Following the addition of the three studies, the pooled prevalence of MM was revised to 9.9% (95% CI: [1.8%–39.7%]) as shown in Table 4. There is more heterogeneity among studies, with an *I* ^**2**^ of 90.4%.

**TABLE 4:** Pooled estimate of MGUS in Sub‐Saharan Africa before and after trim and fill analysis.

Studies	Pooled estimate	95% CI	Number of studies
Before trim and fill analysis	3.1%	0.8%–12.0%	8
After trim and fill analysis	9.9%	1.8%–39.7%	11

### 3.3 Advanced Stage in MM in Sub‐Saharan Africa

Six studies with a combined 12,044 MM patients reported the proportion of MM patients based on either the Durie Salmon Staging and the International staging system, yielding 7347 stage III cases. The pooled prevalence of late presentation was 62.1% (95% CI: 51.3%–71.8%).

Individual study estimates ranged from 48.9% in South Africa (Chili 2023) to 82.9% in Tanzania (Raza 2024), with the largest single contribution from a national‐level cohort in Madagascar (Randrianarisoa 2023), in which 61.0% of patients were staged as DSS III.

Substantial heterogeneity was observed (*I* ^**2**^ = 86.3%; *τ* ^2^ = 0.2711; *p* < 0.0001). Despite this variation, all studies consistently demonstrated a high burden of advanced disease at diagnosis, indicating that late presentation is common in Sub‐Saharan Africa.

## 4 Mortality From MM in Sub‐Saharan Africa

### 4.1 Pooled Mean Duration of Survival (in Months)

The simple pooled mean survival is 34.7 months using sample‐size weighting.

#### 4.1.1 Year Overall Survival

**FIGURE 9:**
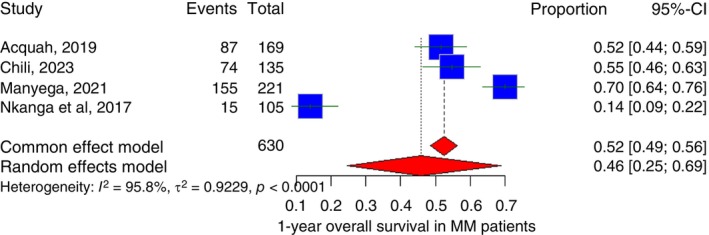
Forest plot of 1‐year overall survival of multiple myeloma in Sub‐Saharan countries.

Only four studies included a 1‐year overall survival percentage. The pooled 1‐year overall survival across all included studies was 46.0% (95% CI: 24.6% to 69.0%) with *I* ^2^ of 95.8% (Figure 9).

#### 4.1.2 Year Overall Survival

**FIGURE 10:**
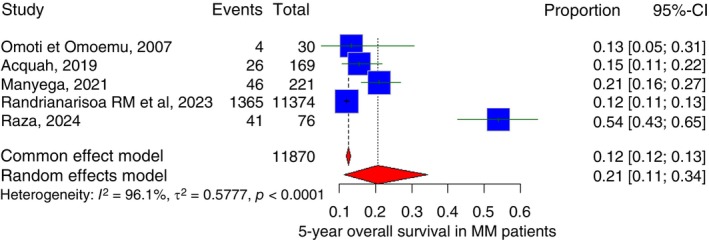
Forest plot of 5‐year overall survival of multiple myeloma in Sub‐Saharan countries.

Five studies included a 5‐year overall survival percentage. The pooled 5‐year overall survival across all included studies was 20.7% (95% CI: 11.4% to 34.5%) with *I* ^2^ of 96.1% (Figure 10).

## 5 Association of Human Immunodeficiency Virus With MM in Sub‐Saharan Africa

**FIGURE 11:**
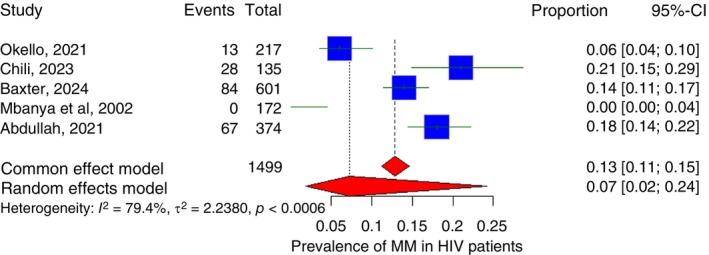
Forest plot showing the prevalence of MM among HIV‐positive patients in Sub‐Saharan Africa.

Only five studies measured the proportion of MM patients who are also HIV positive, with three of the studies from South Africa, one from Uganda and the last one from Cameroon. The pooled prevalence of MM among HIV/AIDS patients in Sub‐Saharan Africa is 7.2% (95% CI: 1.9%–24.2%) with *I* ^2^ of 79.4% (Figure 11). Subgroup analysis pooled prevalence of MM among HIV/AIDS patients in South Africa is 16.6% (95% CI: 13.7%– 20.1%) with *I* ^2^ of 62.1%.

The pooled mean survival duration of studies without a reported significant HIV/AIDS prevalence is 36.7 months while the pooled mean survival duration of studies with a reported significant HIV prevalence is 9.9 months. Tables 5 and 6 show the mean survival duration in individual studies without and with reported HIV prevalence, respectively.

**TABLE 5:** Mean survival duration of studies without a reported HIV/AIDS prevalence in multiple myeloma.

Study	Country	Mean survival duration	HIV/AIDS prevalence in MM
Nnonyelum et al. [19]	Nigeria	7.4	Not reported
Omoti et Omoemu [20]	Nigeria	7	Not reported
Salawu et Durosinmi [21]	Nigeria	1.2	Not reported
Acquah et al. [22]	Ghana	33	Not reported
Raza et al. [32]	Tanzania	18	Not reported
Manyega et al. [26]	Kenya	29	Not reported
Nkanga et al. [27]	DR Congo	Not reported	Not reported
Babatunde et al. [30]	Nigeria	44	Not reported
Randrianarisoa RM et al. [29]	Madagascar	45.5	Not reported
Oelofse et Truter [53]	South Africa	11	Not reported
Dirisu et al. [31]	Nigeria	Not reported	Not reported

**TABLE 6:** Mean survival duration of studies with a reported HIV/AIDS prevalence in multiple myeloma.

Study	Country	Mean survival duration	HIV/AIDS prevalence in MM
Okello et al. [23]	Uganda	2.5	14.7%
Chili et al. [24]	South Africa	18	21.0%
Baxter et al. [25]	South Africa	5.6	14.0%
Mbanya et al. [28]	Cameroon	Not Stated	0.0
Abdullah et al. [33]	South Africa	18	18.0%

## 6 Association of Human Immunodeficiency Virus With MGUS in Sub‐Saharan Africa

No studies measured the proportion of MGUS patients who are also HIV positive. However, two studies, one in South Africa and the other done in Eswatini provided the odds ratio of the association between HIV and MGUS. The pooled odds ratio of HIV/AIDS among MGUS patients in Sub‐Saharan Africa is 1.5 (95% CI 0.7–3.2), indicating that individuals with HIV/AIDS have 1.5 times higher odds of developing MGUS than those without HIV/AIDS in sub Saharan Africa. However this is not statistically significant.

## 7 Association of Renal Failure With MM in Sub‐Saharan Africa

**FIGURE 12:**
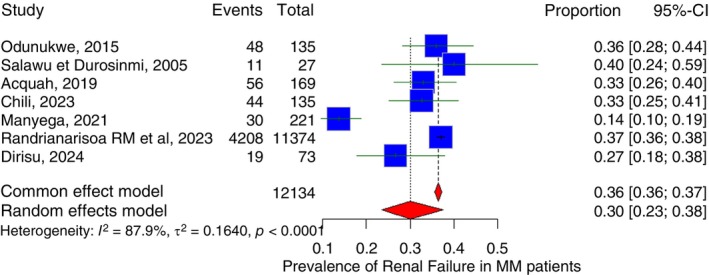
Forest plot showing the prevalence of renal failure among MM patients in Sub‐Saharan Africa.

Seven studies measured the proportion of renal failure in patients with MM in Sub‐Saharan Africa. Three of the studies were from Nigeria, one from Ghana, one from South Africa, one from Kenya and the last from Madagascar. The pooled prevalence of renal failure among MM patients in Sub‐Saharan Africa is 30.1% (95% CI: 23.5%–37.6%) with *I* ^2^ of 87.9% (Figure 12). Subgroup analysis pooled prevalence of renal failure among MM patients in Nigeria is 33.5% (95% CI: 27.8%–39.8%) with *I* ^2^ of 15.1%.

The pooled mean survival duration of studies with a reported significant Renal Failure (taken as equal or greater than 33%) prevalence is 22.5 months while the pooled mean survival duration of studies with a reported non‐significant Renal Failure prevalence (less than 33%) is 24.8 months. Because the included studies reported renal‐failure prevalence using heterogeneous thresholds and definitions, we used a data‐driven approach to create analytically comparable subgroups. The study‐level prevalence of renal failure was dichotomised at the median value (33%), which is a standard, unbiased method of reducing ecological variability and preventing arbitrary thresholding. Median splits are widely used in meta‐epidemiological analyses when continuous study‐level covariates lack standard clinical cut‐offs. Moreover, studies reporting > 30%–33% prevalence of renal failure were classified as ‘high renal‐failure burden’, consistent with established epidemiological thresholds used in large myeloma cohorts. Randrianarisoa et al. (2023) was excluded from the pooled mean survival despite reporting a renal failure prevalence due to extremely high sample size relative to other studies in the meta‐analysis.

## 8 Association of Anaemia With MM in Sub‐Saharan Africa

To evaluate whether anaemia burden influenced survival, we fitted a sample‐size–weighted linear regression model comparing high versus low anaemia prevalence studies as shown in Tables 7 and 8. The estimated mean survival in low anaemia‐burden studies was 24.4 months (SE = 6.9). Studies with higher anaemia burden showed a non‐significant 5.5‐month shorter survival (*β* = −5.50; SE = 8.31; *p* = 0.54). Anaemia group explained only 8% of the between‐study variation in survival (*R* ^2^ = 0.08), and overall model fit was poor (*F* (1, 5) = 0.44, *p* = 0.54). These findings indicate no detectable study‐level association between anaemia burden and mean survival.

**TABLE 7:** Characteristics of included studies reporting haemoglobin levels and survival among multiple myeloma patients in Sub‐Saharan Africa.

First author, year	Sample size	Mean Hb (g/dL)	SD (g/dL)	Estimated anaemia (%)	Mean survival (months)	Anaemia group
Salawu et Durosinmi [21]	27	7.87	2.47	80.6	1.2	High Anaemia
Nnonyelum et al. [19]	135	8.4	2.1	77.7	7.4	High Anaemia
Acquah [22]	169	8.97	9.63	54.3	33	High Anaemia
Abdullah [33]	374	9.7	2.8	54.3	18	High Anaemia
Raza [32]	76	9.77	3.11	52.9	18	Low Anaemia
Manyega [26]	221	10	8.52	50	29	Low Anaemia
Omoti et Omoemu	30	10.3	2.7	45.6	7	Low Anaemia

**TABLE 8:** Subgroup analysis of mean survival by anaemia burden (Hb < 10 g/dL vs. ≥ 10 g/dL).

Anaemia prevalence	Number of studies	Sample (*N*) size	Pooled survival (months)	Unweighted mean survival (months)
High anaemia burden (Hb < 10g/dL)	4	705	18.9	14.9
Low anaemia burden (Hb ≥ 10g/dL)	3	327	24.4	18

## 9 Association of Hypercalcemia With MM in Sub‐Saharan Africa

Six studies measured the proportion of MM patients with hypercalcemia (serum calcium greater than 2.75 mmol/L). The pooled prevalence of MM patients with hypercalcemia in Sub‐Saharan Africa is 23.9% (95% CI: 15.1%–39.7%) with *I* ^2^ of 97.0%.

The pooled mean survival duration of studies with a reported hypercalcemia (level greater than 2.75 mmol/L) is 22.5 months while the pooled mean survival duration of studies without a reported hypercalcemia level is 12.7 months, likely reflecting differences in diagnostic capacity, follow‐up completeness and health‐system resource availability rather than a direct biological effect. Randrianarisoa et al. (2023) was excluded despite reporting a hypercalcemia prevalence due to an extremely high sample size relative to other studies in the meta‐analysis.

## 10 Discussion

We estimated pooled prevalences of 7.8% for MM and 3.1% for MGUS, with Central and Southern Africa showing higher burdens than West and East Africa. In contrast, population‐based studies from Europe and North America typically report MGUS prevalences of around 3%–6% among adults aged ≥ 50 years, while MM remains much less common [62, 63]. The relatively low MGUS prevalence in our meta‐analysis probably reflects under‐ascertainment, as most included studies were hospital‐based and conducted in settings with limited access to immunofixation and serum free light chain assays, sparse population screening and long delays from symptom onset to MM diagnosis [22, 64].

Consistent with global literature, our findings confirm a disproportionately higher burden of MM among Black populations [10, 15, 62, 65, 66, 67, 68, 69, 70]. Compared with landmark data from Olmsted County in the United States, where MGUS prevalence was 3.2% in those over 50 years of age and progression risk was about 1% annually, our pooled MGUS prevalence was similar, yet MM prevalence was higher [62]. This discrepancy may reflect under‐detection of MGUS in SSA, where diagnostic limitations mean patients are only diagnosed at symptomatic MM stage [62, 69]. Moreover, there are only a few studies currently available on MGUS in Sub‐Saharan Africa. Another study in the United States showed that MGUS prevalence is significantly higher among Blacks than Whites (0.88% vs. 0.22%), with an age‐adjusted prevalence ratio (ASIR) of 3.0 [10, 65]. At the global level, Australasia consistently reports the highest MM incidence rates (ASIR 5.3–5.8/100,000), while Western Europe bears the largest absolute patient numbers [15, 66]. By contrast, Western Africa has among the lowest reported ASIR (0.81 [0.39–1.66]) [67]. Nonetheless, this estimate for Western Africa should be interpreted with caution, given the paucity of high‐quality incidence studies from the region.

The higher prevalences of MM reported in studies conducted between 2011 and 2025 may be linked to advances in diagnostic and surveillance techniques, as well as the growing burden of risk factors such as obesity, toxic exposures from urbanisation and other non‐communicable diseases [68, 70]. However, this apparent increase may not necessarily reflect a true rise in MM prevalence; rather, it could largely stem from improvements in research methodologies and reporting practices. The Covid 19 pandemic also seems to have slowed down MM diagnosis, possibly due to reduced hospital visits during lockdowns [71].

The pooled mean survival for MM in Sub‐Saharan Africa, based on this meta‐analysis, was 34.7 months, with a 1‐year overall survival and 5‐year overall survival of 46.0% and 20.7%, respectively. In contrast, survival outcomes in high‐income countries have improved substantially over the past two decades. For example, a 2025 report from a mid‐level Spanish hospital found a median survival of 38.8 months (3.2 years) among patients without access to ASCT, slightly higher than the 34.7 months observed in Sub‐Saharan Africa [2]. Similarly, data from the US Surveillance, Epidemiology and End Results (SEER) program for 2016–2020 showed a 5‐year relative survival of approximately 55.6%, far exceeding the 20.7% pooled estimate for the Sub‐Saharan region [72]. Several factors may explain these disparities, including delayed diagnosis resulting from late presentation, limited access to essential diagnostic tools such as serum protein electrophoresis, immunofixation and serum free light‐chain testing, as well as restricted availability of cytogenetics and fluorescence in situ hybridization (FISH) [6, 7]. Additional contributors include a scarcity of hematopathology expertise and low clinician awareness of early or atypical manifestations of MM [6, 7]. In addition, restricted access to novel therapies and ASCT, coupled with socioeconomic barriers such as low income and inadequate healthcare infrastructure, further contribute to the lower survival and higher mortality observed in Sub‐Saharan Africa [73, 74].

HIV emerged as an important modifier, with MM prevalence over 15% among people living with HIV and survival reduced to less than 7 months compared to nearly 28 months in HIV‐negative patients. This aligns with prior work showing HIV‐driven immune dysregulation accelerates plasma cell disorders and complicates diagnosis [75, 76]. Importantly, HIV shifts the onset of MGUS and MM to younger ages, compounding the clinical and social burden in SSA [9, 75, 76].

Approximately one‐third of Sub‐Saharan African patients with MM had renal failure (30.1%, 95% CI 23.5%–37.6%). This is broadly comparable to reports from high‐income settings, where 20%–50% of newly diagnosed myeloma patients have renal impairment and around 5%–10% require dialysis at presentation [77, 78]. Ho et al. reported renal impairment in 36% of patients in the Australia and New Zealand Myeloma Registry, while Courant et al. found a prevalence of 24.6% in a French population‐based registry, and a Brazilian cohort described kidney insufficiency in over 40% of newly diagnosed cases [78, 79, 80]. Although the prevalence appears similar to high‐income settings, the drivers of kidney injury in Sub‐Saharan Africa are different, with many patients presenting years after symptom onset, often presenting in the advanced stages. Moreover, the additional renal stress imposed by advanced‐stage MM therefore occurs against a backdrop of pre‐existing renal vulnerability, which likely contributes to the higher observed prevalence of renal failure [81, 82].

The poorer outcomes observed are likely attributable to multiple systemic challenges, including underdeveloped diagnostic capacity, delayed presentation, absence of routine screening programmes and limited access to contemporary therapeutic options. Together, these factors highlight the urgent need for strengthened health infrastructure, improved diagnostic pathways and equitable access to effective treatments to address the burden of plasma cell disorders in Sub‐Saharan Africa. Such efforts would not only clarify the true MGUS–MM relationship in the region but also improve survival outcomes and advance equity in myeloma care globally.

### 10.1 Limitations

Several key limitations of our study should be acknowledged when interpreting the findings. The primary limitation of our study is the small number of available studies from Sub‐Saharan Africa, particularly for MGUS, HIV‐associated MGUS and survival estimates, which limited the power of statistical analyses and precluded more extensive subgroup analyses.

The included studies exhibited substantial heterogeneity in both methodology and population characteristics. In addition, we observed evidence of publication bias, with funnel plot asymmetry and Egger's regression test suggesting that studies reporting higher prevalence rates were more likely to be published. Although non‐parametric trim‐and‐fill methods were applied to adjust for this bias, the revised pooled estimates remain hypothetical and may not fully reflect true prevalence.

Diagnostic limitations also remain a critical challenge in Sub‐Saharan Africa. Restricted access to advanced hematopathology services and low clinical awareness likely result in substantial under‐diagnosis of MGUS, particularly its asymptomatic precursor forms. This under‐detection skews the literature toward symptomatic MM, as many patients are diagnosed only at advanced stages of the disease.

Finally, the lack of comprehensive data on key variables, such as disease stage, treatment regimens and comorbidities (notably HIV/AIDS) restricted the scope of subgroup analyses. Although we observed striking differences in survival by HIV status, the small sample size renders this finding suggestive rather than definitive.

### 10.2 Strengths

This review has several notable strengths. To our knowledge, it is the first systematic review and meta‐analysis to synthesise the epidemiology, clinical characteristics and outcomes of MM in Sub‐Saharan Africa. The study followed the PRISMA 2020 guidelines, applied a registered research quality protocol (the Joanna Briggs Institute tool) and used robust methodological approaches, including duplicate screening, data extraction and risk‐of‐bias assessment to minimise reviewer bias.

The inclusion of studies across multiple regions of Sub‐Saharan Africa provides a broader perspective on the burden of MM and highlights regional differences in presentation, diagnostic access and outcomes. By incorporating data on MGUS, HIV‐associated MM and survival outcomes, this review also expands the understanding of contextual factors that may shape disease expression in African populations. Beyond prevalence, we synthesised HIV‐associated MM and survival outcomes, generating pooled estimates that highlight clinically important regional variation.

## 11 Conclusion

Our study demonstrated a comparatively high prevalence of MM in Sub‐Saharan Africa, aligning with evidence that these conditions are more common in Black populations than in White populations. However, the prevalence of MGUS relative to MM was low in Sub‐Saharan Africa, which could be attributed to delayed presentation and poor diagnostic tools. This contributes to clarifying the epidemiology of MGUS and MM in a region where data have historically been sparse and fragmented.

Mortality from MM appears disproportionately higher in Sub‐Saharan Africa compared with other regions. HIV infection may act as a catalyst for more aggressive disease phenotypes and remains a complicating factor in the management of both MGUS and MM in this setting. Renal Failure prevalence in MM is relatively comparable to those in high‐income settings, with one‐third of MM patients coming down with Renal Failure.

Overall, the study underscores the need for improved surveillance, diagnostic infrastructure and equitable treatment access. The findings provide a foundation for policymakers, clinicians, and researchers to prioritise resources and develop regionally appropriate interventions to improve MM outcomes in Sub‐Saharan Africa.

## Funding

The authors have nothing to report.

## Ethics Statement

This study is a systematic review and meta‐analysis of previously published literature and did not involve the collection of new data from human participants or animals. Therefore, ethical approval was not required, and no approval was sought from an Institutional Review Board (IRB) or Ethics Committee. All data used in this study were extracted from peer‐reviewed articles that had already obtained appropriate ethical approvals from their respective institutions.

## Consent

The authors have nothing to report.

## Conflicts of Interest

The authors declare no conflicts of interest.

## Data Availability

The data that support the findings of this study are available from the corresponding author upon reasonable request.
